# Venous Tumor Thrombus Level in Renal Cell Carcinoma: Impact on Surgical and Oncologic Outcomes

**DOI:** 10.3390/cancers18111801

**Published:** 2026-06-01

**Authors:** Zuzanna Korbecka, Beata Jabłońska, Robert Król

**Affiliations:** 1 Department of General, Vascular and Transplant Surgery, Medical University of Silesia, 40-027 Katowice, Poland; zuzanna.korbecka@sum.edu.pl (Z.K.); robertk@hot.pl (R.K.); 2 Department of Digestive Tract Surgery, Medical University of Silesia, 40-752 Katowice, Poland

**Keywords:** renal cell carcinoma, tumor thrombus, inferior vena cava, thrombectomy, prognosis

## Abstract

Background: Renal cell carcinoma (RCC) with venous tumor thrombus (VTT) represents a clinically challenging entity requiring complex surgical management. The prognostic significance of tumor thrombus level remains controversial, with conflicting evidence regarding its impact on perioperative and oncologic outcomes. Methods: This narrative review summarizes the current literature on the relationship between VTT level and surgical complexity, perioperative outcomes, and long-term oncologic results in patients undergoing radical nephrectomy with thrombectomy. Results: Higher levels of tumor thrombus are consistently associated with increased surgical complexity, including longer operative time, greater blood loss, and higher rates of perioperative complications. However, the impact of thrombus level on oncologic outcomes remains inconsistent across studies. Most contemporary evidence suggests that VTT level alone is not an independent predictor of survival, whereas tumor biology, nodal and distant metastatic staging, and histological features, plays a more significant role. Conclusions: While tumor thrombus level is a key determinant of surgical planning and technical difficulty, its prognostic value for long-term survival appears limited. Clinical decision-making should therefore integrate both anatomical and biological tumor characteristics to optimize patient outcomes.

## Simple Summary

Renal cell carcinoma with venous tumor thrombus is a rare but complex clinical condition that often requires extensive surgery. This review evaluates whether the level of tumor thrombus influences surgical difficulty and long-term outcomes. While higher thrombus levels are associated with more technically demanding procedures and increased perioperative risk, their impact on survival remains unclear. Current evidence suggests that tumor biology and metastatic status are more important determinants of prognosis than thrombus level alone. These findings highlight the importance of individualized treatment strategies based on both anatomical and biological factors.

## 1. Introduction

Renal cell carcinoma (RCC) is the most common type of kidney cancer, accounting for approximately 2–3% of adult malignancies [1]. It is characterized by a distinctive propensity to invade the venous system, extending from the renal parenchyma into the renal vein and, in a subset of cases, the inferior vena cava (IVC). Venous tumor thrombus (VTT) occurs in approximately 4–15% of patients, representing a clinically significant challenge that impacts both surgical planning and prognosis [2,3].

For patients without metastatic disease, radical nephrectomy with thrombectomy is the standard curative approach. Nevertheless, these procedures carry substantial perioperative risk, with reported mortality ranging from 5% to 15%, and complication rates increasing with the cranial extent of the thrombus. These challenges have motivated the development of risk-adapted strategies, including preoperative thrombus reduction through systemic therapy or selective interventional techniques, as well as approaches aimed at minimizing surgical invasiveness when feasible. Such strategies aim to reduce morbidity and mortality without compromising oncologic control, reflecting a broader shift toward individualized, multidisciplinary management in RCC patients with venous involvement [2,3,4].

The extent of venous involvement is most commonly classified using the Mayo Clinic classification, which stratifies tumor thrombus into levels 0–IV based on its cranial extent. While this system was originally developed to guide surgical planning, its prognostic significance remains an area of ongoing debate.

This narrative review summarizes current evidence regarding the association between tumor thrombus level and perioperative, oncologic, and survival outcomes in RCC.

## 2. Classifications of Venous Tumor Thrombus

There are two common classifications of the anatomical extent of thrombus: Neves Zincke and Mayo Clinic classifications.

The anatomical tumor thrombus location has direct implications for surgical complexity, need for vascular or cardiothoracic support, and perioperative risk [5,6,7,8].

### 2.1. Neves Zincke Classification

The Neves Zincke classification distinguishes: level I: tumor thrombus confined to renal vein, level II: thrombus extending into the infrahepatic IVC, level III: thrombus extending into the retrohepatic IVC, and level IV: thrombus extending into the right atrium [5,6].

### 2.2. Mayo Clinic Classification

Mayo Clinic classification defines tumor thrombus levels as follows: level 0: tumor thrombus confined to the renal vein, level I: thrombus extending <2 cm into the IVC above the renal vein, level II: thrombus extending >2 cm into the IVC but below the hepatic veins, level III: thrombus extending above the hepatic veins but below the diaphragm, level IV: thrombus extending above the diaphragm or into the right atrium [7,8]. The above mentioned classifications are compared in Table 1.

### 2.3. American Joint Committee on Cancer (AJCC) TNM Staging System (8th Edition)

The current American Joint Committee on Cancer (AJCC) TNM staging system (8th edition) incorporates tumor thrombus level into T-stage classification, with T3a designating renal vein or segmental branch involvement, T3b indicating IVC extension below the diaphragm, and T3c representing supradiaphragmatic IVC involvement or IVC wall invasion. This staging system was revised in 2010 (7th edition) specifically to differentiate renal vein-only involvement (T3a) from IVC thrombus (T3b/T3c), reflecting the hypothesis that thrombus level carries independent prognostic significance [9,10].

**Table 1:** Comparison of Neves Zincke and Mayo Clinic classifications and American Joint Committee on Cancer (AJCC) TNM staging system (8th edition).

Neves–Zincke Classification	Description	Mayo Clinic Classification	Description
0	No equivalent	0	Thrombus limited to the renal vein
I	Thrombus extending into the renal vein	I	Thrombus extending into the IVC <2 cm above the renal vein
II	Thrombus reaching the infrahepatic IVC	II	thrombus extending >2 cm above the renal vein but below hepatic veins
III	Thrombus extending into the retrohepatic/suprahepatic IVC below the diaphragm	III	Thrombus extending into the intrahepatic/suprahepatic IVC below the diaphragm
IV	Thrombus extending above the diaphragm or into the right atrium	IV	Thrombus extending above the diaphragm or into the right atrium
**American Joint Committee on Cancer (AJCC) TNM staging system (8th edition)**			
T1	≤7 cm, limited to the kidney		
T1a	≤4 cm		
T1b	>4 cm but ≤7 cm		
T2	>7 cm, limited to the kidney		
T2a	>7 cm but ≤10 cm		
T2b	>10 cm		
T3	Extends into major veins or perinephric tissues, but not into the ipsilateral adrenal gland and not beyond Gerota’s fascia		
T4	Tumor invades beyond Gerota’s fascia (including contiguous extension into the ipsilateral adrenal gland)		
N0	No regional lymph node metastasis		
N1	Metastasis in regional lymph node(s)		
M0	No distant metastasis		
M1	Distant metastasis (e.g., bones, brain, lungs, liver, noncontiguous adrenal gland)		
Stage I	T1, N0, M0		
Stage II	T2, N0, M0		
Stage III	T1 or T2 with N1, M0; OR T3, N0/N1, M0		
Stage IV	T4, any N, M0; OR Any T, any N, M1		

IVC; inferior vena cava.

## 3. Search Strategy and Study Selection

This narrative review was conducted to summarize and critically discuss the literature published over the past decade regarding the relationship between venous tumor thrombus level and both surgical and oncological outcomes in patients with renal cell carcinoma undergoing radical nephrectomy with thrombectomy. Relevant publications were identified through a non-systematic search of the PubMed database using combinations of predefined keywords and Medical Subject Headings (MeSH), including “renal cell carcinoma with tumor thrombus,” “tumor thrombus level,” “nephrectomy with thrombectomy,” “surgical outcomes,” “postoperative complications,” and “oncological outcomes.” The review focused primarily on English-language full-text articles published between 2016 and 2025. Priority was given to studies considered most relevant to the topic, including retrospective observational studies, systematic reviews, and meta-analyses addressing surgical or oncological outcomes associated with tumor thrombus level. Case reports and publications not directly related to the scope of the review were not considered.

## 4. Literature Review

### 4.1. Tumor Thrombus Level and Perioperative Outcomes

#### 4.1.1. Surgical Complexity According to Tumor Thrombus Level

Recent literature consistently demonstrates that increasing venous tumor thrombus (VTT) level in renal cell carcinoma is associated with greater surgical complexity and less favorable perioperative outcomes. Across multiple retrospective series and systematic reviews, patients with higher-level thrombi generally experience longer operative times, greater intraoperative blood loss, increased transfusion requirements, prolonged intensive care unit and hospital stays, and higher rates of perioperative complications [8,9,10,11]. These findings are biologically plausible, as advanced thrombus extension often necessitates more extensive vascular dissection, hepatic mobilization, inferior vena cava (IVC) control, and, in selected cases, cardiopulmonary bypass (CPB), extracorporeal circulation, or complex vascular reconstruction [12]. Despite the increased operative burden associated with advanced thrombus levels, contemporary high-volume centers continue to report acceptable perioperative mortality rates, emphasizing the importance of surgical expertise and multidisciplinary perioperative management [5,8,9,10].

#### 4.1.2. Surgical Strategies and Technical Considerations

The extent of thrombus invasion substantially influences the surgical strategy. According to the systematic review by Lardas et al. [12], level I thrombi usually require only minor modifications of standard nephrectomy techniques, whereas level II thrombi generally necessitate vascular control above and below the thrombus, including control of the contralateral renal vein and lumbar tributaries. In contrast, levels III–IV frequently require extensive caval and hepatic dissection, advanced vascular techniques, and occasionally CPB or deep hypothermic circulatory arrest [12]. Although CPB has historically been associated with prolonged operative time, coagulopathy, and greater blood loss, some studies suggest that, in experienced centers, its use does not necessarily worsen perioperative outcomes [13]. Alternative strategies, including non-CPB approaches, venovenous bypass, and Foley-catheter-assisted thrombectomy, have also demonstrated feasibility and acceptable safety profiles in selected patients [14,15,16,17,18].

#### 4.1.3. Operative Time, Blood Loss, and Transfusion Requirements

Several studies have identified higher thrombus level as one of the strongest predictors of operative complexity. Liu et al. [19] demonstrated that patients with small renal tumors but high-level thrombi had significantly longer operative times, greater blood loss, higher transfusion requirements, and more frequent postoperative complications than patients with larger tumors and lower thrombus extension, suggesting that thrombus level may influence surgical difficulty more strongly than primary tumor size itself. Similar associations between VTT level and operative time, blood loss, transfusion requirements, intensive care utilization, and prolonged hospitalization were subsequently confirmed in multiple retrospective cohorts [20,21,22,23,24,25,26,27,28,29,30,31,32,33,34,35,36,37,38,39,40,41]. Studies focusing on advanced surgical procedures, including visceral resections or IVC reconstruction, further emphasized the technical challenges associated with higher-level thrombi [23,28,40].

#### 4.1.4. Perioperative Complications and Mortality

Most studies also reported increasing rates of severe perioperative complications with advancing thrombus level [21,34,39]. For example, Ralla et al. [21] observed major complications in 8.4% of patients with level II thrombi compared with 29.5% in patients with level IV disease, while Dell’Oglio et al. [34] demonstrated significantly higher rates of Clavien–Dindo grade ≥3 complications among patients with level III–IV thrombi. However, this association has not been observed consistently across all series. Several investigators reported no statistically significant relationship between thrombus level and postoperative complication rates or perioperative mortality [6,27,33,36,38,40]. These discrepancies may reflect differences in institutional experience, sample size, patient selection, surgical techniques, perioperative management protocols, and the proportion of highly advanced cases included in individual cohorts.

#### 4.1.5. Additional Prognostic and Technical Factors

Additional factors beyond thrombus level may also influence perioperative outcomes. Recent studies highlighted the importance of aggressive histologic variants, IVC wall invasion, the need for vascular reconstruction, extracorporeal circulation, metastatic disease, and patient comorbidity burden [24,28,33,40]. In particular, Lewis et al. [28] demonstrated that aggressive histologic variants were associated with more adherent thrombi and more frequent IVC wall invasion requiring complex reconstruction, although perioperative outcomes remained acceptable in experienced centers. Similarly, studies evaluating elderly patients suggested that age alone may not significantly worsen perioperative outcomes when surgery is performed in appropriately selected patients [24].

#### 4.1.6. Neoadjuvant Therapy and Future Perspectives

Growing interest has also focused on neoadjuvant therapy (NAT) as a strategy to reduce thrombus burden before surgery. A recent meta-analysis by Gu et al. [2], incorporating data from studies by Okamura et al. [29], Tanaka et al. [30], Cai et al. [31], and Field et al. [32], suggested that NAT may reduce thrombus level in a subset of patients and facilitate less extensive surgery. Several included studies demonstrated reductions in operative time and intraoperative blood loss following NAT, while complication rates remained comparable to those observed in patients undergoing upfront surgery [2,29,30,31,32]. Nevertheless, the authors emphasized that NAT may also introduce additional adverse events and costs, and further studies are required to identify patients most likely to benefit from this approach [2].

#### 4.1.7. Summary

Overall, current evidence supports VTT level as an important determinant of surgical complexity and perioperative risk in patients with renal cell carcinoma undergoing nephrectomy with thrombectomy. At the same time, advances in surgical techniques, vascular reconstruction, perioperative management, and multidisciplinary care have substantially improved outcomes, even among patients with extensive caval involvement [2,5,8,9,10].

### 4.2. Tumor Thrombus Level and Oncologic Outcomes (Overall Survival and Cancer-Specific Survival)

#### 4.2.1. Overall Prognostic Significance of Tumor Thrombus Level

The relationship between venous tumor thrombus (VTT) level and long-term oncologic outcomes in renal cell carcinoma (RCC) remains controversial. Early studies suggested progressively worse survival with increasing thrombus extension; however, more recent analyses have produced inconsistent results. Several retrospective and multicenter studies demonstrated that patients with thrombus limited to the renal vein generally achieve better overall survival (OS) and cancer-specific survival (CSS) than those with inferior vena cava (IVC) involvement [6,42,43]. In contrast, differences between lower (I–II) and higher (III–IV) IVC thrombus levels are often modest and frequently fail to reach statistical significance [18,20,24,25,38,39,42,43]. Overall, contemporary evidence suggests that thrombus level alone may reflect disease extent but does not consistently predict survival independently of other clinicopathological variables.

#### 4.2.2. Studies Supporting the Prognostic Value of Thrombus Level

Several studies reported worse oncologic outcomes with increasing thrombus extension. Rodriguez et al. [42], in one of the largest cohorts, demonstrated that higher thrombus level independently predicted worse cancer-specific survival after adjustment for other prognostic variables. Similarly, Bokka et al. [21], Dell’Oglio et al. [34], and Navratil et al. [37] observed poorer survival outcomes among patients with higher-level thrombi, particularly when comparing renal vein thrombus with extensive caval involvement. Our previous study [6] also demonstrated significantly shorter OS among patients with IVC extension compared with those with thrombus confined to the renal vein. These findings support the concept that advanced venous extension may reflect more aggressive disease biology and increased metastatic potential.

#### 4.2.3. Studies Questioning the Independent Prognostic Role of Thrombus Level

In contrast, many contemporary studies failed to confirm thrombus level as an independent predictor of survival. Large retrospective and multicenter analyses demonstrated that, after adjustment for other adverse clinicopathological features, VTT level often loses prognostic significance [25,27,35,38,39,43]. For example, Shiff et al. [43] found no significant differences in recurrence-free survival (RFS), CSS, or OS across thrombus levels 0, I–II, and III–IV, even after multivariable adjustment. Similarly, Chen et al. [25], Miura et al. [40], Crisafi et al. [38], Chao et al. [42], and Shiff et al. [43] reported comparable long-term survival between lower and higher thrombus levels. In several studies, variables reflecting systemic disease burden or tumor biology—including metastatic disease, lymph node involvement, sarcomatoid differentiation, performance status, inflammatory markers, or invasion of adjacent structures—were stronger predictors of survival than thrombus level itself [27,35,42].

These discrepancies among studies likely result from substantial heterogeneity in patient populations, inclusion criteria, tumor biology, surgical management, and follow-up duration. Some cohorts included predominantly nonmetastatic patients, whereas others involved a large proportion of advanced or metastatic disease [20,33,38]. Differences in institutional experience and evolving perioperative management may additionally contribute to variations in survival outcomes. Furthermore, several studies grouped thrombus levels differently, which complicates direct comparisons between cohorts [6,39,42].

#### 4.2.4. Impact of Tumor Biology and Additional Prognostic Factors

Recent evidence increasingly suggests that long-term oncologic outcomes are driven more strongly by tumor biology and systemic disease status than by the anatomical extent of venous involvement alone. Multiple studies identified metastatic disease, nodal involvement, tumor size, sarcomatoid differentiation, collecting system invasion, inflammatory markers, and performance status as major determinants of prognosis [27,35,42]. Rodriguez et al. [42] additionally demonstrated the prognostic significance of microscopic venous wall invasion, while Lewis et al. [28] highlighted the importance of aggressive histologic variants associated with more invasive vascular behavior.

Importantly, several studies demonstrated acceptable long-term outcomes even in selected patients with advanced level III–IV thrombi when surgery was performed in experienced centers [19,23,24,27]. Five-year OS rates in contemporary cohorts generally range from approximately 40% to 60% [25,27,37,42], supporting the role of aggressive surgical management in appropriately selected patients.

#### 4.2.5. Systemic Therapy and Evolving Treatment Strategies

Growing interest has also focused on the role of systemic therapy in RCC-VTT patients. Sandberg et al. [44] demonstrated that preoperative systemic therapy was associated with significantly improved OS and CSS in patients with metastatic RCC-VTT, whereas postoperative systemic therapy did not significantly improve outcomes. In nonmetastatic patients, systemic treatment did not confer additional survival benefit. These findings suggest that patient selection and disease stage are critical when considering multimodal treatment strategies.

Similarly, Huang et al. [45] demonstrated a substantial survival advantage associated with upfront surgery compared with conservative management in RCC-VTT patients, reporting a median OS of 51.7 months vs. 13.4 months, respectively. Most surgically treated patients experienced either no or only minor perioperative complications, supporting the continued role of radical nephrectomy with thrombectomy as the cornerstone of treatment whenever technically feasible.

#### 4.2.6. Summary

Overall, current evidence suggests that although VTT level correlates with disease extent and surgical complexity, its independent prognostic significance for long-term survival remains uncertain. Studies reporting poorer survival with advanced thrombus extension coexist with analyses demonstrating no independent effect of VTT level after adjustment for other clinicopathological variables [6,21,25,27,34,35,37,38,39,42,43]. These conflicting findings likely reflect differences in tumor biology, metastatic burden, patient selection, and institutional expertise rather than the anatomical thrombus level alone. Contemporary evidence increasingly supports the concept that systemic disease characteristics and aggressive tumor biology are stronger determinants of survival than venous extension itself [27,35,42,43]. Further prospective multicenter studies are required to better define the prognostic role of VTT level and optimize risk stratification in patients with RCC and venous tumor thrombus.

Characteristics of the above-described studies are summarized in Table 2.

## 5. Impact of Concomitant Adverse Features

The above mentioned conflicting evidence regarding the prognostic significance of VTT level may be partially explained by the coexistence of additional adverse clinicopathological features that more strongly reflect tumor biology and systemic disease burden.

While tumor thrombus level has traditionally been considered a primary prognostic indicator in RCC, accumulating evidence demonstrates that concomitant adverse pathologic and clinical features often exert a more profound impact on oncologic outcomes than thrombus extent alone. Multiple large-scale studies have consistently identified sarcomatoid differentiation as one of the most powerful independent predictors of survival, with hazard ratios ranging from 2.0 to 3.7 for OS and CSS [2,25,46]. The presence of tumor necrosis has similarly emerged as a significant adverse feature, independently associated with decreased survival across multiple cohorts [25,47,48]. Perinephric fat invasion represents another critical prognostic factor, with studies demonstrating its association with both CSS and RFS, leading some investigators to propose refinement of the T3 classification to incorporate this feature [2,25,49]. Lymph node involvement consistently demonstrates strong prognostic significance, with hazard ratios exceeding 2.0 in most series, while metastatic disease at presentation substantially diminishes survival regardless of thrombus level [2,6,25]. Additional adverse features include high Fuhrman or International Society of Urological Pathology (ISUP) nuclear grade, collecting system invasion, adrenal gland invasion, and large tumor size (typically >10 cm), all of which have been validated as independent predictors in multivariate analyses [2,6,10,35,47,50]. Notably, several studies have found that when these adverse pathologic features are present, the prognostic impact of thrombus level itself becomes less significant or even non-significant in multivariate models, suggesting that tumor biology and local disease extent may supersede anatomic thrombus extension in determining patient outcomes [25,46]. These findings underscore the importance of comprehensive pathologic assessment and risk stratification beyond thrombus level alone when counseling patients and planning treatment strategies for RCC-VTT.

**Figure 1:**
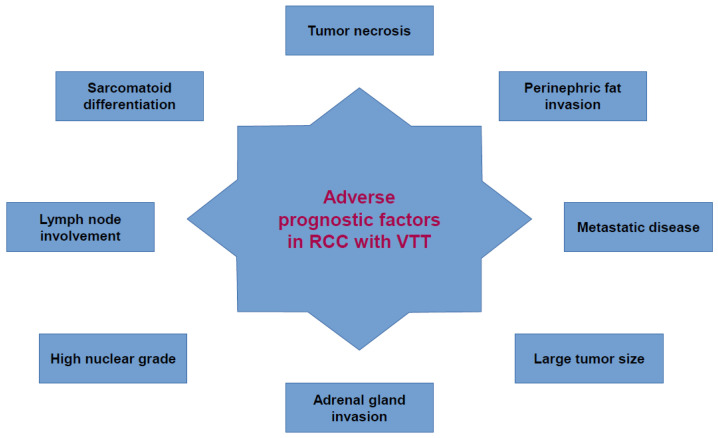
Adverse prognostic factors in renal cell carcinoma with venous tumor thrombus (RCC, Renal Cell Carcinoma; VTT, Venous Tumor Thrombus).

All above-mentioned prognostic factors are presented in Figure 1.

## 6. Predictive Nomograms Including Tumor Thrombus Level

### 6.1. Mayo Clinic 2026 Nomogram for Metastasis-Free Survival

The most recently developed and extensively validated nomogram was proposed by Roberson et al. [51] at the Mayo Clinic. It was based on a cohort of 532 patients who underwent nephrectomy with venous thrombectomy for non-metastatic RCC between 2000 and 2021. The model is intentionally simplified and includes only four variables: thrombus level according to the Mayo classification (0–IV), tumor necrosis, sarcomatoid differentiation, and pathological lymph node stage (pN). The nomogram demonstrated strong predictive performance, achieving an Area Under Curve (AUC) of 0.74 in both the training and internal validation cohorts. External validation in two independent institutional datasets yielded AUC values of 0.71 and 0.68. Decision curve analysis further indicated that the model provides a net clinical benefit across threshold probabilities ranging from 0.33 to 0.80, supporting its application in selecting candidates for adjuvant Pembrolizumab therapy. Within this cohort, the estimated overall 5-year metastasis-free survival rate was 41.3%. The distribution of thrombus levels was as follows: Level 0—52.3%, level I—12.4%, level II—21.8%, level III—6.6%, and level IV—7.0% [51].

### 6.2. Abel Nomogram for Recurrence

The Abel nomogram, created by Abel et al. [52], was derived from a multi-institutional cohort of 636 patients treated at five centers and is designed to estimate the risk of recurrence after surgery for non-metastatic RCC with tumor thrombus. Independent prognostic variables included tumor size, body mass index, preoperative hemoglobin below the lower limit of normal, thrombus level, perinephric fat invasion, and non-clear cell histology. The overall estimated 5-year RFS was 49%. When stratified by the number of risk factors, 5-year RFS rates were 77% for patients with no risk factors, 53% for those with one, 47% for two, and 20% for patients with more than two risk factors. The model showed consistent predictive performance in both the development and validation cohorts, with AUC values of 0.726 and 0.724, respectively. Moreover, it demonstrated better predictive accuracy than several general RCC prognostic systems, including the UCLA Integrated Staging System (UISS) (AUC 0.726 vs. 0.595, *p* = 0.001), the tumor stage, size, grade, and necrosis score (SSIGN) (AUC 0.713 vs. 0.612, *p* = 0.04), and the Sorbellini nomogram (AUC 0.709 vs. 0.638, *p* = 0.02) [52].

### 6.3. Chinese Nomograms

Li et al. [48] created a prognostic nomogram based on a cohort of 228 Chinese patients. Their analysis identified several independent predictors of overall survival: hemoglobin levels below the lower limit of normal (HR 1.73), the presence of sarcomatoid differentiation (HR 3.67), perinephric fat invasion (HR 1.80), non–clear cell histological subtype (HR 2.74), and the presence of metastases at the time of surgery (HR 1.71). The model demonstrated good predictive performance with a concordance index (C-index) of 0.77. Interestingly, tumor thrombus level was not retained in the final multivariate model, indicating that pathological characteristics had greater prognostic value than the anatomical extent of the thrombus in this cohort [48].

In an another study, Zhang et al. [53] developed a nomogram to predict progression-free survival using data from 199 patients. Independent adverse prognostic factors included Fuhrman grade 4 (HR 1.92), papillary RCC histology (HR 3.02), perinephric fat invasion (HR 1.54), and sarcomatoid differentiation (HR 2.97), whereas adjuvant therapy showed a protective effect (HR 0.32). In this cohort, PFS rates at 1, 3, and 5 years were 78.4%, 45.4%, and 30.0%, respectively, with a median progression-free survival of 41.0 months [53].

### 6.4. Primary Tumor Score Nomogram

In 2025, Zhang et al. [54] proposed a new prognostic model introducing a primary tumor score based on tumor necrosis and the morphology of the tumor thrombus. This score showed stronger prognostic performance than traditional parameters such as the level of IVC tumor thrombus and tumor size. The final nomogram incorporated several variables: the primary tumor score, presence of distant metastases, non–clear cell histological subtype, sarcomatoid differentiation, preoperative anemia grade, and the American Society of Anesthesiologists (ASA) Physical Status Classification System level. The model demonstrated good predictive accuracy, with a concordance index (C-index) of 0.77 and AUC values of 0.80, 0.81, and 0.78 for predicting 1-, 2-, and 3-year OS, respectively. These results suggest that tumor biology and morphological characteristics may have greater prognostic significance than the anatomical extent of tumor thrombus alone [54].

### 6.5. Gu Nomogram

The Gu nomogram was developed by Gu et al. (2017) [55] using data from 185 patients with renal cell carcinoma and venous tumor thrombus who underwent surgical resection between 2006 and 2016. After a median follow-up period of 30.2 months, several variables were identified as independent predictors for OS, including histological subtype, invasion of the collecting system, the presence of metastases at the time of surgery, the De Ritis ratio (AST/ALT), and serum albumin levels. The model demonstrated moderate predictive accuracy with a concordance index (C-index) of 0.75. Importantly, the VTT level was not included in the final multivariable model, suggesting that biological tumor characteristics and metabolic markers may be more informative predictors of survival than the anatomical extent of the thrombus [55].

### 6.6. Zhao Nomogram

The Zhao et al. [56] analyzed outcomes in 138 patients with non-metastatic renal cell carcinoma accompanied by venous tumor thrombus, with particular attention to the role of deep invasive tumor thrombus (DITT). The presence of DITT was linked to increased surgical complexity, including longer operative times, greater intraoperative blood loss, higher transfusion requirements, and a higher incidence of postoperative complications. Multivariable analysis identified several independent predictors of overall survival: sarcomatoid differentiation, tumor thrombus invasion, low hemoglobin levels, and pathological subtype. The model demonstrated good predictive performance, achieving a concordance index of 78.8% (95% CI: 71.2–86.4%). Unlike many earlier models, this nomogram highlights the depth of vessel wall invasion by the thrombus—reflecting thrombus invasiveness—rather than its anatomical level as the key thrombus-related prognostic factor [56].

### 6.7. Clinical Nomograms Utility and Limitations

Available nomograms reveal several consistent observations. First, sarcomatoid differentiation and tumor necrosis repeatedly appear as strong independent prognostic factors across most models, frequently demonstrating hazard ratios greater than approximately 2.5–3.7. Second, when tumor thrombus level is included in multivariable analyses, its prognostic impact is generally less pronounced than that of these key pathological characteristics. Third, easily obtainable laboratory markers, such as preoperative hemoglobin and the De Ritis ratio (aspartate aminotransferase/alanine aminotransferase ((AST/ALT)), can provide additional prognostic information prior to surgery. In terms of performance, most nomograms show moderate to good discriminative ability, with concordance indices typically ranging from 0.75 to 0.79. However, a common limitation is that many models have undergone only internal validation, most often using bootstrap resampling techniques. Notable exceptions include the nomograms developed at the Mayo Clinic and the Abel nomogram, both of which have also been externally validated in independent patient cohorts. This highlights the ongoing need for prospective studies and broader external validation before these tools can be widely implemented in routine clinical practice [48,51,52,53,54,55,56].

All above-described nomograms are summarized in Table 3.

## 7. Clinical Implications of the Tumor Thrombus Level and Future Directions

The evolving understanding of tumor thrombus level has important implications for clinical practice. While VTT remains essential for surgical planning and risk stratification, comprehensive pathologic assessment incorporating sarcomatoid differentiation, tumor necrosis, nuclear grade, lymph node status, and metastatic disease is critical for accurate prognostic estimation and treatment decision-making [25,43,44,46,49]. Contemporary prognostic nomograms for RCC with tumor thrombus increasingly incorporate these multifactorial assessments rather than relying on thrombus level alone [46,49,50,52]. For patient counseling, clinicians should emphasize that surgical outcomes and long-term survival are determined more by tumor biology than by anatomic thrombus extent, though higher thrombus levels do confer increased perioperative risk and surgical complexity. The decision to proceed with surgery should be based on comprehensive risk assessment incorporating performance status, comorbidities, tumor characteristics, and patient preferences, rather than thrombus level in isolation [19,25,41,48,51,52].

The role of neoadjuvant systemic therapy may be particularly relevant for patients with high-level thrombus, as 35% of patients in the neoadjuvant axitinib for reducing the extent of VTT in RCC with venous invasion (NAXIVA) trial experienced reduction in tumor thrombus level with neoadjuvant axitinib, and contemporary immune checkpoint inhibitor plus tyrosine kinase inhibitor (ICI-TKI) combinations demonstrate even higher response rates [2,56,57,58]. The NAXIVA trial (Phase II Neoadjuvant Study of Axitinib for Reducing Extent of Venous Tumour Thrombus in Renal Cancer with Venous Invasion, NCT03494816) was a phase II, single-arm, multicentre study that evaluated the use of neoadjuvant axitinib, a vascular endothelial growth factor receptor (VEGFR)-targeted tyrosine kinase inhibitor (TKI), for reducing RCC-associated VTT [58]. Whether NAT can convert high-level thrombi to lower levels and thereby reduce surgical complexity while improving oncologic outcomes remains an active area of investigation. Combination therapy with ICIs has emerged as the first-line treatment for metastatic RCC. Given the aggressive behavior of RCC with VTT, patients may benefit from neoadjuvant ICI therapy to help stabilize the disease and potentially decrease tumor size prior to surgery. However, data on the surgical safety of this approach remain limited, with only a small number of cases reported from referral centers. Furthermore, clinical trials investigating neoadjuvant ICIs have not consistently prioritized surgical safety as a primary endpoint, nor have they provided detailed evaluations of how neoadjuvant immunotherapy may influence surgical complexity [2,58,59,60]. According to Khene et al. [60], preoperative immunotherapy appears to be safe and feasible in patients with RCC and IVC thrombus undergoing radical nephrectomy and thrombectomy, as it does not seem to increase postoperative morbidity despite longer operative times. However, larger prospective studies with longer follow-up are required to validate these results [60].

Future research should focus on integrating molecular and genomic markers with anatomic and pathologic features to develop more precise prognostic models. The identification of distinct molecular signatures associated with tumor thrombus formation and progression may enable better patient stratification and personalized treatment approaches [61]. Additionally, prospective studies evaluating the impact of neoadjuvant and adjuvant therapies specifically in the tumor thrombus population are needed to optimize multimodal treatment strategies [62].

## 8. Biological Considerations—Molecular and Immunologic Features of Tumor Thrombus

Emerging molecular and immunologic evidence suggests that venous tumor thrombi constitute a biologically distinct tumor compartment rather than a simple intravascular extension of the primary lesion. Comparative analyses have demonstrated differences in immune cell composition between primary tumors and thrombus tissue [61,62,63,64,65]. For instance, Liss et al. (2019) [63] reported significant heterogeneity in immune infiltration patterns in renal cell carcinoma with venous tumor thrombus, including altered proportions of macrophages, natural killer cells, and other immune populations in thrombus samples compared with the primary tumor [63].

In addition, transcriptomic profiling further supports the presence of a distinct tumor ecosystem within the thrombus. Using single-cell RNA sequencing, Shi et al. (2022) [64] identified a complex multicellular microenvironment within vena caval tumor thrombi characterized by specific stromal and immune cell populations, enhanced extracellular matrix remodeling, and altered intercellular signaling pathways relative to the primary tumor [64].

Genomic studies have also demonstrated marked intrapatient heterogeneity between these compartments. An integrative genomic analysis by Wang et al. (2020) [65] revealed that a substantial proportion of somatic mutations were unique either to the primary tumor or to the tumor thrombus, suggesting partially independent clonal evolution of the intravascular component [65]. The study by The authors (2020) [65] performed an integrative genomic analysis of clear cell renal cell carcinoma using whole-exome sequencing and transcriptomic profiling in a large Chinese patient cohort. Tumors associated with venous tumor thrombus demonstrated distinct molecular characteristics, including a higher frequency of mutations in genes such as BAP1 and SETD2, as well as increased genomic instability. The authors also observed substantial intratumoral heterogeneity between primary tumors and thrombus tissue, suggesting that tumor thrombi may arise through partially independent clonal evolution and represent a biologically distinct component of the disease [65]. Differences in immune checkpoint expression have also been observed. Wang et al. [65] reported variability in PD-L1 expression and immune cell density between primary tumors and associated venous thrombi, findings that may have implications for systemic immune responses and oncologic outcomes [66]. More recently, single-cell transcriptomic analyses by Tao et al. (2025) [66] demonstrated enrichment of macrophage populations and exhausted CD8^+^ T cells within tumor thrombi, together with activation of signaling pathways associated with tumor cell migration, survival, and vascular interaction [67]. Shapiro et al. (2023) [67] emphasized that RCC is characterized by a highly immunogenic yet frequently immunosuppressive microenvironment, with prominent infiltration of T lymphocytes, macrophages, and myeloid-derived suppressor cells that can promote tumor progression and immune evasion. These interactions, together with variable expression of immune checkpoint molecules such as PD-1/PD-L1, are considered key determinants of disease progression and response to immunotherapy [67].

Collectively, these findings support the concept that tumor thrombi represent a distinct biological niche with unique molecular and immunologic characteristics. This perspective further reinforces the hypothesis that intrinsic tumor biology and metastatic potential—rather than the cranial extent of the thrombus alone—may play a dominant role in determining oncologic outcomes [63,64,65,66].

## 9. Discussion

A consistent finding across studies is the association between increasing thrombus level and surgical complexity. Higher-level thrombi extending into the suprahepatic IVC or right atrium require more extensive operative strategies, including hepatic mobilization, complex vascular control, and in selected cases cardiopulmonary bypass (CPB). Consequently, operative time, blood loss, transfusion requirements, and complication rates increase with cranial thrombus extension. However, outcomes from high-volume centers indicate that acceptable perioperative results can still be achieved even in advanced disease, provided that multidisciplinary expertise is available [5,8,9,10,20,28,33].

Despite these observations, the evidence base is limited by substantial methodological weaknesses. The majority of studies are retrospective, single-center series with small sample sizes and strong selection bias [6,18,19,25,33,36,40]. Patients are often highly selected for surgical eligibility, limiting generalizability. In addition, long study periods introduce temporal heterogeneity in surgical technique, perioperative care, and systemic therapy use.

Marked methodological heterogeneity further limits comparability between studies. Differences in thrombus classification systems (e.g., Mayo vs. Neves–Zincke), perioperative definitions, and reporting of complications significantly affect outcome interpretation [12,20,21,34]. This heterogeneity reduces the strength of pooled conclusions and contributes to variability in reported results.

A key unresolved issue is whether VTT level represents an independent prognostic factor. While univariable analyses frequently demonstrate worse survival with increasing thrombus extent, this association often disappears in multivariable models [6,25,33,36,40,43]. These findings suggest that thrombus level may function more as a surrogate marker of overall tumor burden and aggressive tumor biology rather than as an independent biological determinant of prognosis. Importantly, discrepancies between studies may be partially explained by differences in multivariable adjustment strategies. Studies in which the prognostic significance of thrombus level disappears after adjustment generally incorporate a broader range of pathological confounders, including sarcomatoid differentiation, tumor necrosis, lymph node involvement, and metastatic burden. In contrast, studies that continue to identify thrombus level as an independent predictor of survival often adjust for fewer clinicopathological variables, which may overestimate the prognostic impact of thrombus extent alone [42]. These methodological differences likely contribute substantially to the conflicting results reported in the literature and further highlight the limitations of the currently available retrospective evidence.

The role of minimally invasive and robotic-assisted surgery in RCC with VTT remains evolving. While robotic-assisted techniques have been increasingly reported, their application is currently limited to highly selected patients, primarily those with lower-level thrombi (I–II). Robotic procedures are mainly performed in patients with lower-level thrombi (Mayo I–II), lower tumor burden, and more favorable anatomical conditions, whereas open surgery was more frequently applied in cases with advanced thrombus extension (Mayo III–IV) [68,69,70,71,72]. This imbalance in baseline characteristics likely explains the more favorable perioperative outcomes reported in robotic cohorts. Available evidence suggests that minimally invasive approaches may be feasible in experienced high-volume centers; however, management of advanced thrombi (Mayo III–IV) remains technically demanding because of the need for complex vascular control, caval reconstruction, liver mobilization, and, in selected cases, extracorporeal circulation. Consequently, widespread adoption of robotic techniques for advanced RCC-VTT remains limited. Furthermore, the currently available evidence is derived predominantly from small retrospective series with substantial selection bias and without robust comparative oncologic data. Therefore, although robotic surgery may represent a promising option for carefully selected patients with lower-level thrombi treated at experienced high-volume centers, open surgery remains the gold standard for most patients with RCC and venous tumor thrombus, particularly for Mayo level III and above. While a few highly specialized centers have reported successful fully robotic management of selected Mayo III–IV thrombi and complex cases involving inferior vena cava wall invasion, most centers are currently unable to achieve the degree of vascular control and liver mobilization required for these procedures robotically, and such approaches cannot yet be considered standard practice. Peng et al. [70] highlighted the importance of liver mobilization for retrohepatic IVC exposure during level II–III robot-assisted IVC thrombectomy and suggested that caudate lobectomy may facilitate vascular control and improve perioperative outcomes in complex cases. Huang et al. [71] reported that robotic surgery was mainly applied in selected patients with low-level thrombi, whereas open surgery remained preferred for technically demanding cases, including large tumors, retrohepatic thrombi, and severe adhesions. Similarly, Amparore et al. [72] demonstrated that only a minority of patients undergoing minimally invasive surgery presented with Mayo III–IV thrombi, reflecting substantial selection bias. Although MIS was associated with favorable perioperative outcomes, robust comparative oncologic evidence remains lacking, and robotic surgery for Mayo III–IV thrombi should currently be reserved for exceptional cases in highly experienced centers [12,28,40,69,70,71].

The integration of systemic therapy into the perioperative management of RCC-VTT remains an active area of investigation. With increasing use of immune checkpoint inhibitors and targeted therapies, neoadjuvant strategies have been explored as a means to downstage thrombus burden. Evidence suggests that neoadjuvant therapy may reduce thrombus level or tumor size in selected patients and potentially facilitate less extensive surgery with reduced intraoperative blood loss [2,29,30,31,32]. However, responses are heterogeneous, and predictors of treatment efficacy remain poorly defined. Importantly, oncologic benefits in terms of survival have not been consistently demonstrated across studies, and most available data are derived from small observational cohorts with variable treatment regimens. Therefore, neoadjuvant therapy should currently be considered investigational and reserved for selected patients in specialized centers [2,58,59,60].

The management of RCC-VTT requires an individualized, multidisciplinary approach. Surgical decision-making should not rely solely on thrombus level but should integrate tumor biology, metastatic status, patient comorbidities, and institutional expertise. For example, in elderly patients with significant comorbidities but no metastatic disease and level IV thrombus, the decision to proceed with surgery must balance potential long-term oncologic benefit against substantial perioperative risk and expected quality of recovery. In this context, factors such as sarcomatoid differentiation, tumor burden, and likelihood of achieving complete resection may be more informative than anatomical thrombus extent alone [25,35,42].

**Figure 2:**
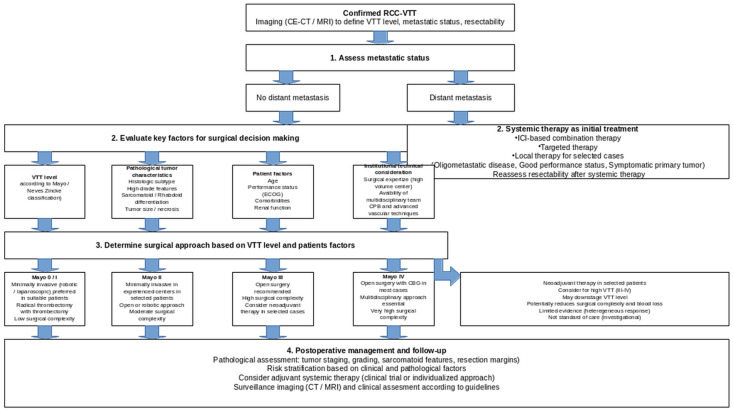
Clinical decision flowchart for management of renal cell carcinoma (RCC) with VTT (venous tumor thrombus). CE-CT, contrast-enhanced computed tomography; MRI, magnetic resonance imaging; ICI, immune checkpoint inhibitor; ECOG, Eastern Cooperative Oncology Group; CPB, cardiopulmonary bypass.

Multidisciplinary management remains essential for optimizing outcomes. Close collaboration between urologists, vascular and hepatobiliary surgeons, anesthesiologists, and intensive care specialists is critical. High-volume referral centers consistently demonstrate improved perioperative outcomes and lower complication rates, reinforcing the importance of centralization of care [5,8,9,10,20,28,33]. A clinical decision flowchart is presented in Figure 2.

## 10. Future Directions

Despite growing interest in VTT in RCC, significant gaps in knowledge persist. Most available evidence is derived from retrospective, single-center studies with heterogeneous patient populations, limiting the ability to establish the independent prognostic role of thrombus level [18,25,43]. Future research should prioritize well-designed prospective and multicenter studies to improve the quality and generalizability of the evidence.

A key direction involves integrating anatomical parameters, such as thrombus level, with tumor biology. Emerging data suggest that molecular and genomic features may reflect tumor aggressiveness better than anatomical staging alone [21,24]. Therefore, combining clinicopathological variables with molecular profiling could lead to more accurate risk stratification models.

In this context, artificial intelligence (AI) and radiomics represent promising tools. Advanced imaging analysis may allow for non-invasive characterization of tumor thrombus, assessment of vascular wall invasion, and prediction of surgical complexity and oncologic outcomes. Machine-learning-based models may further enhance predictive accuracy by integrating multidimensional data, including imaging, clinical, and molecular features [34].

Moreover, future studies should explore the role of systemic and neoadjuvant therapies in downstaging tumor thrombus and improving resectability. The integration of targeted therapies and immunotherapy into multimodal treatment strategies remains an area of active investigation [32,33].

Finally, there is a need for robust external validation of existing nomograms and the development of universally applicable predictive tools. Such models should be easy to implement in clinical practice and capable of supporting personalized decision-making in patients with RCC and VTT [30,31].

## 11. Conclusions

The level of venous tumor thrombus in renal cell carcinoma is a critical factor influencing surgical strategy and perioperative risk. Higher thrombus levels are consistently associated with increased technical complexity, longer operative times, and greater intraoperative morbidity. However, current evidence does not support a consistent independent association between thrombus level and long-term oncologic outcomes. Instead, tumor biology, nodal and distant metastatic staging, grading, and histological features, appears to play a more decisive role in determining survival.

From a clinical perspective, these findings underscore the importance of comprehensive preoperative assessment and multidisciplinary decision-making. Surgical management should be tailored not only to the anatomical extent of the thrombus but also to the underlying tumor characteristics. Future studies should focus on integrating clinical, pathological, and molecular factors to improve risk stratification and guide personalized treatment strategies in this complex patient population.

## 12. Clinical Key Points

- Tumor thrombus level is a critical determinant of surgical planning and perioperative risk, but its role as a prognostic marker is limited.
- Aggressive surgical management remains justified in well-selected patients without distant metastases, regardless of thrombus level.
- Prognostic models should integrate thrombus level with pathologic, clinical, and molecular factors to improve risk stratification.
- From a clinical perspective, tumor thrombus level remains crucial for surgical planning but should not be used as the sole prognostic factor. Multidisciplinary evaluation and individualized treatment strategies are essential to optimize outcomes in patients with RCC and venous tumor thrombus.

## Figures and Tables

**Table 2:** Summary of included studies with prognostic variables in RCC with venous tumor thrombus.

First Author	Year	Country	Study Design	Patients (n)	VTT Level	% Metastasis	Median FU	MVA Performed	VTT Level Independent Predictor	Perioperative Findings	Oncologic Outcomes
Liu et al. [19]	2019	China	Retrospective	67	0–IV	NR	14 mo	Yes	No	↑ operative time, blood loss	No effect on CSS
Nini et al. [20]	2021	Italy	Retrospective	19	Mayo III	NR	NR	No	NA	Morbidity high	2y OS 60%
Chen et al. [25]	2021	China	Retrospective	121	0–IV	26%	24 mo	Yes	No	↑ surgery time, LOS	No OS impact
Shiff et al. [43]	2021	Multicenter	Retrospective	228	0–IV	0%	21.2 mo	Yes	No	Comparable periop outcomes	No survival impact
Ishiyama et al. [24]	2021	Japan	Retrospective	123	I–IV	40.7%	25.2 mo	Yes	No	No diff complications	No OS/CSS effect
Bokka et al. [22]	2022	India	Retrospective	34	I–IV	NR	58 mo	Yes	Yes	↑ complexity	Worse survival
Gonzalez et al. [23]	2021	USA	Retrospective	18	Advanced	40–50%	24 mo	No	NA	Feasible surgery	Favorable OS
Ralla et al. [21]	2022	Germany	Retrospective	61	II–IV	24.6%	23 mo	Yes	No	↑ complexity	Metastasis strongest factor
Horynecka et al. [6]	2022	Poland	Retrospective	102	I–IV	2.94%	21.5 mo	Yes	No	NA	Tumor biology > VTT
Fang et al. [26]	2023	China	Retrospective	55	0–IV	30.9%	291 days	Yes	NA	↑ blood loss, LOS	Worse function
Nagamoto et al. [27]	2023	Japan	Retrospective	55	I–IV	~16%	44.2 mo	Yes	No	↑ operative time	No complication difference
Lewis et al. [28]	2024	USA	Retrospective	403	I–IV	27% No AHV 42% AHV	NR	Yes	No	AHV ↑ complexity	Worse prognosis with AHV
Gu et al. [2]	2024	Multicenter	Meta-analysis	204	I–IV	Variable	NR	Yes	NA	NAT reduces thrombus	Comparable outcomes
Hanquiez et al. [33]	2024	France	Retrospective	42	I–IV	28.1%	NR	Yes	No	No VTT effect on complications	No survival effect
Dell’Oglio et al. [34]	2024	Italy	Retrospective	40	I–IV	30%	1070 days (I–II) 482 days (I–II)	Yes	NA	↑ complications in high VTT	Worse OS in III–IV
Faria-Costa et al. [35]	2024	Portugal	Retrospective	64	0–III	14.1%	NR	Yes	No	↑ operative burden	Biology stronger predictor
Khalil et al. [36]	2025	Egypt	Retrospective	14	I–IV	NR	19.5 mo	No	NA	Low mortality	No association
Navratil et al. [37]	2025	Czech	Retrospective	164	I–IV	12.1%	NR	Yes	Yes	↑ operative time	Worse DFS
Crisafi et al. [38]	2025	Italy	Retrospective	39	I–IV	36%	49 mo	Yes	No	High mortality IV	No OS difference
Lee et al. [39]	2025	Korea	Retrospective	287	0–IV	28.2%	NR	Yes	No	↑ complications	No OS difference
Miura et al. [40]	2025	Japan	Retrospective	29	I–IV	6.9%	31 mo	No	No	↑ CPB use	Similar survival

RCC, renal cell carcinoma; VTT, Venous tumor thrombus; OS, overall survival; CSS, cancer-specific survival; DFS, disease-free survival; NAT, neoadjuvant therapy; AHV, aggressive histologic variants; CPB, cardiopulmonary bypass; FU, follow-up; mo, months; NA, not analyzed; NR, not reported.

**Table 3:** Nomograms used in RCC with venous tumor thrombus.

First Author et al.	Year	Country	Nomogram Name	Description	Use
Roberson et al. [51]	2026	USA	Mayo Clinic Metastasis-Free Survival Nomogram	Simplified model including VTT level (Mayo 0–IV), tumor necrosis, sarcomatoid differentiation, and pN stage; externally validated	Prediction of metastasis-free survival and selection for adjuvant immunotherapy
Abel et al. [52]	2014	Multicenter	Abel Recurrence Nomogram	Model incorporating tumor size, BMI, hemoglobin, VTT level, perinephric fat invasion, and histology	Prediction of recurrence-free survival after surgery
Li et al. [48]	2020	China	Chinese RCC-VTT OS Nomogram	Model based on hemoglobin, sarcomatoid differentiation, perinephric fat invasion, histology, and metastases; VTT level excluded in final model	Prediction of overall survival
Zhang et al. [53]	2021	China	RCC-VTT Progression-Free Survival Nomogram	Model including Fuhrman grade, histology, perinephric fat invasion, sarcomatoid differentiation, and adjuvant therapy	Prediction of progression-free survival
Zhang et al. [54]	2025	China	Primary Tumor Score Nomogram	Model introducing a novel tumor score based on necrosis and thrombus morphology combined with clinical variables	Prediction of overall survival
Gu et al. [55]	2017	China	Gu RCC-VTT Nomogram	Model based on histology, collecting system invasion, metastases, De Ritis ratio, and albumin; VTT level not included	Prediction of overall survival
Zhao et al. [56]	2022	China	DITT-Based Nomogram	Model emphasizing deep invasive tumor thrombus (DITT) along with hemoglobin, histology, and sarcomatoid differentiation	Prediction of overall survival and surgical risk

VTT, venous tumor thrombus; RCC, renal cel carcinoma; BMI, body mass index; deep invasive tumor thrombus.

## Data Availability

No new data were created or analyzed in this study.
